# Bone-patellar tendon-bone allograft reconstruction for peri-patellar tendon sarcomas: case series

**DOI:** 10.1186/s40064-015-1510-9

**Published:** 2015-11-26

**Authors:** Jungo Imanishi, Damien Grinsell, Peter F. M. Choong

**Affiliations:** Department of Orthopaedics, St. Vincent’s Hospital Melbourne, 41 Victoria Parade, Fitzroy, 3065 Australia; Department of Orthopaedic Oncology and Surgery, Saitama Medical University International Medical Center, 1397-1 Yamane, Hidaka, Saitama 350-1298 Japan; Department of Plastic Surgery, St. Vincent’s Hospital Melbourne, 41 Victoria Parade, Fitzroy, 3065 Australia; Department of Surgery, St. Vincent’s Hospital Melbourne, University of Melbourne, Level 2, Clinical Sciences Building, 29 Regent Street, Fitzroy, VIC 3065 Australia

**Keywords:** Soft tissue sarcoma, Reconstruction, Patellar tendon, Knee extension mechanism, Allograft

## Abstract

**Introduction:**

Reconstruction after wide resection for a sarcoma involving the knee extensor mechanism is challenging even if the tumor is small.

**Case description:**

We report on four consecutive peri-patellar tendon sarcomas treated similarly at a single institution. Histological diagnoses were synovial sarcoma (two cases), clear cell sarcoma and extraskeletal Ewing’s sarcoma (one case each). Follow-up periods after surgery were 18–67 months. All cases underwent pre-operative radiotherapy and subsequent surgery. After preoperative radiotherapy and wide resection including the patellar tendon, bone-patellar tendon-bone allograft was fixed to the residual patella and tibial tuberosity with screws and a cable wire. Soft tissue and skin defect over allograft was covered by free antero-lateral thigh flap. Post-operatively, the operated knee was splinted straight for at least 6 weeks, and then range-of-motion exercise was gradually introduced. Except for one case with a proximal tibial stress fracture 5 months post-operatively, no complication was observed. Both bone–bone junctions between allograft and residual bones were united within 1 year after surgery. At the latest clinical follow-up, all the patients had satisfactory functions with Musculoskeletal Tumor Society score of 28–30 out of 30 points and virtually full range of motion.

**Discussion and evaluation:**

This case series is the first to report bone-patellar tendon-bone allograft for reconstruction after tumor resection with joint preservation and with satisfactory clinical outcomes.

**Conclusions:**

Bone-patellar tendon-bone allograft reconstruction with vascularized flap reconstruction is a viable option for peri-patella tendon sarcomas.

**Electronic supplementary material:**

The online version of this article (doi:10.1186/s40064-015-1510-9) contains supplementary material, which is available to authorized users.

## Background

The mainstay of soft tissue sarcoma treatment for cure is surgical resection with or without adjuvant therapy, but functional loss after wide resection sometimes can be crucial. If a sarcoma involves or abuts on the knee extension mechanism, functional reconstruction after resection can be complex and challenging as in patellar tendon ruptures after total knee replacement (TKR). Several single case reports of peri-patellar tendon sarcomas reconstructed by a recycled bone (Muramatsu et al. 2010), bone-tendon grafts (Peyser and Makley 1996; Osanai et al. 2008; Nakashima et al. 2012), a muscle flap (Machens et al. 2005) and a tendon graft (Fukui et al. 1999), have been reported, mainly from Japan where the use of allograft is fairly limited.

Allograft is a well-established reconstruction option for musculoskeletal defect, including reconstruction for bone sarcomas and disruption of the extensor mechanism related to TKR (Rosenberg 2012; Hornicek et al. 1998). Although allograft can be associated with a high rate of infection and fracture if used massively for bony reconstruction, it has several advantages including simple procedure, less invasiveness to patients with no necessity of harvesting tendon, and the preservation of the whole specimen for accurate histological analysis when used for musculoskeletal tumors. In our surgical team, the combination of patellar tendon allograft with bony attachment on both ends and vascularized flap coverage has been used since 2009 because this reconstruction was expected to offer satisfactory limb functions with oncological safety, less procedural complexity, and minimal complications.

The aim of this study is to highlight a novel reconstruction option using bone-patellar tendon-bone (BPTB) allograft for peri-patellar tendon sarcomas, including post-operative management, and to reveal its advantages and disadvantages compared to previous reports.

## Case description

This is a case series of 4 consecutive peri-patella-tendon sarcomas surgically treated with the same allograft and flap reconstruction at our institution between 2009 and 2014. Prior to this study, the institutional review board approval was obtained (HREC-A: QA 117/14). Patients were all male aged 26–72 years at the time of surgery, with follow-up periods of 18–67 (mean 40.8) months. Histological diagnoses were synovial sarcoma (two cases), clear cell sarcoma and extraskeletal Ewing’s sarcoma (one case each). Three out of the four cases had undergone unplanned excision externally with inadequate margin before referral. All the cases underwent pre-operative radiotherapy comprised of external beam 50.4 Gy in divided doses over 28 sessions, and one extraskeletal Ewing’s sarcoma case received neo-adjuvant chemotherapy. All cases achieved wide margin according to the Enneking classification (Enneking et al. 1980), with microscopically negative margin. Patient background and clinical results are summarized in Table 1. Post-operatively, the patients were managed similarly but modified from the preceding case(s) in terms of the period of keeping the operated knee straight in splint (Table 2). Clinical review of each case followed our follow-up protocol: every 3 months for the first 2 years for intermediate- or high-grade soft tissue sarcomas after surgery, with physical examination, chest computed tomography for lung metastasis surveillance and X-ray to evaluate bone union at junctions between the implanted allograft and host bones. Because of the screws and cable wire, magnetic resonance imaging study was not routinely performed for local recurrence surveillance in this case series.

**Table 1:** Summary of 4 cases in this series

No.	Age, sex	Diagnosis	Tumor location	Follow-up	Functions	Complication
1	26, M	Synovial sarcoma	Infrapatellar region	67 months	MSTS score: 30/30 ROM: 0°–130° No extension lag	None
2	56, M	Clear cell sarcoma	Infrapatellar region	48 months	MSTS score: 30/30 ROM: 0°–145° Extension lag < 5°	None
3	28, M	Ewing’s sarcoma	Infrapatellar region	30 months	MSTS score: 30/30 ROM: 0°–135° Extension lag < 5°	None
4	72, M	Synovial sarcoma	Abutting on the proximal tibia	18 months	MSTS score: 28/30 ROM: 0°–10°–115° Extension lag < 5°	Stress fracture

*M* male, *MSTS* musculoskeletal tumor society, *ROM* range of motion

**Table 2:** Summary of post-operative management in this series

	ROM	Weight bearing, activity
1 Immediately after surgery	Operated knee kept straight in splint	FWB in splint with crutches
2 6–12^a^ weeks after surgery	Gentle ROM exercise, passive and active, started	FWB without crutch, gradually removing splint/brace
3 6 weeks after (2)	Progressive ROM allowed	FWB without splint/brace, no sports activity
4 1 year after surgery	No restriction	No restriction

*ROM* range of motion, *FWB* full weight bearing

^a^The duration differed from case to case

### Patient 1

**Fig. 1:**
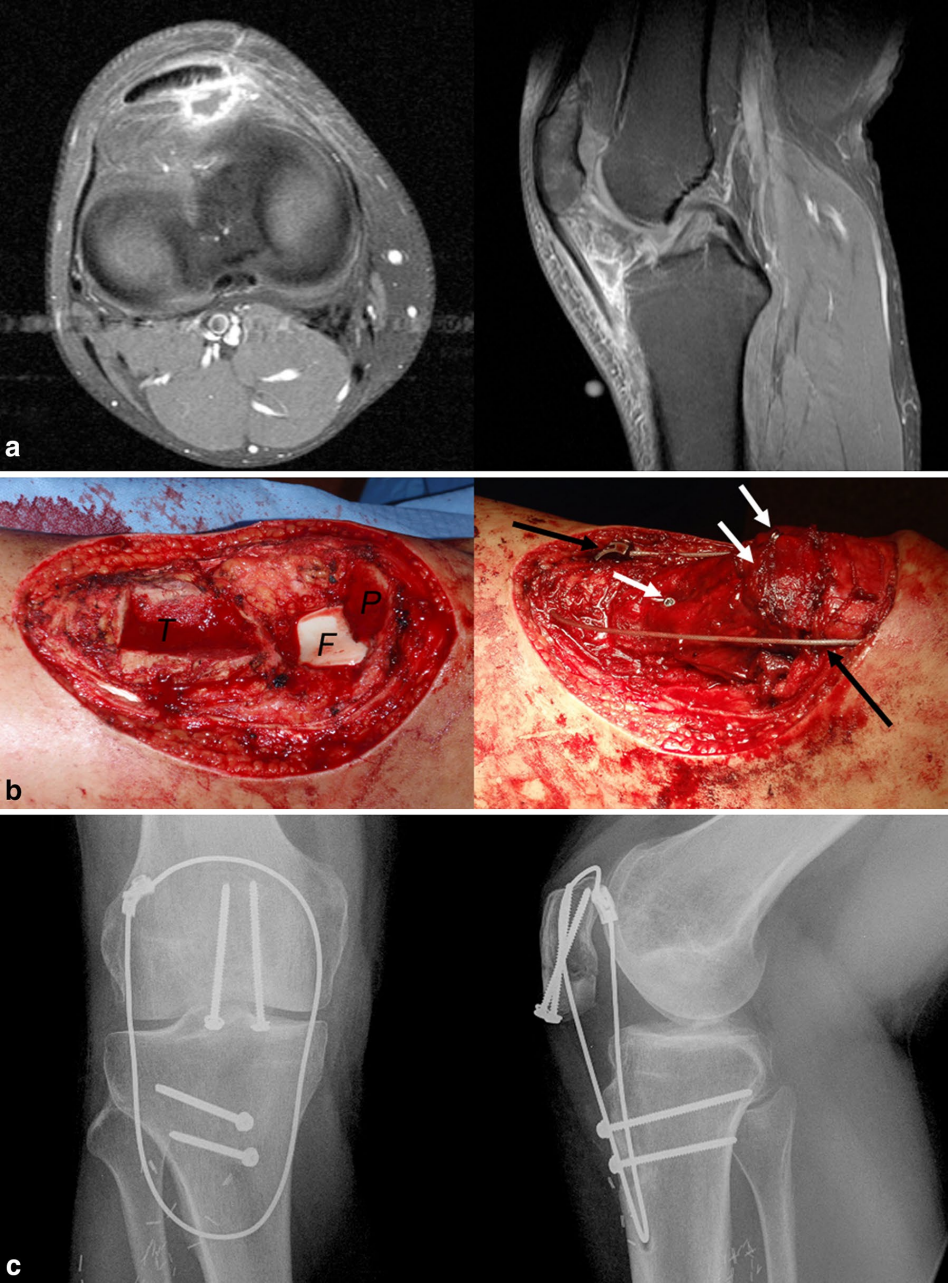
Radiologic studies and photographs of patient 1. **a** T1 gadolinium-enhanced magnetic resonance images at the first presentation show inflammation in the infrapatellar fat pad after unplanned excision. **b** *Photographs* show intra-operative field before and after allograft reconstruction. *White arrows* and *black arrows* point screws and a cable wire system, respectively. **c** Both bone–bone junctions between allograft and residual bones were united at 11 months after surgery. *T* tibia, *F* femoral joint surface, *P* patella

A 25-year-old man was referred after excision of a painful 2-cm nodule in the left infrapatellar region. Pathological diagnosis was synovial sarcoma (Fig. 1a). Because of positive margin, pre-operative radiotherapy and subsequent re-excision were performed. The whole patellar tendon, infrapatellar fat pad, inferior one-third of the patella, and the tibial tuberosity were removed together with 6 × 12 cm of the skin including previous operation scar. Using an oblique osteotomy, nearly the whole original patellar-femoral joint surface of the patella was preserved. Following resection, BPTB allograft was harvested and shaved to fit the osteotomy sites. Then, the allograft was fixed to the residual patella and proximal tibia with screws and a stainless steel Dall-Miles 2-mm Cable and Sleeve Set (Stryker Australia Pty., Ltd.) tightened at 30° knee flexion (Fig. 1b). The soft tissue defect was covered with a free ipsilateral antero-lateral thigh (ALT) flap.

**Fig. 2:**
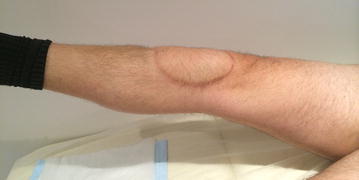
A photograph of the operated knee 4 years post-operatively (patient 1). No extension lag of the operated knee was observed

Post-operative course was uneventful without wound complication or infection. The operated knee was kept straight in splint for the first 3 months, and then gentle passive and active range-of-motion (ROM) exercise was started. Full weight bearing (FWB) was tolerated immediately after surgery but only in the setting of using crutches for the first 6 weeks. Three months after surgery, progressive ROM exercise and FWB without splint were allowed. Both bone–bone junctions were united 11 months post-operatively (Fig. 1c). After both bone–bone junctions were united, sports activity, including fun skiing, was permitted. At 67 months after surgery, the patient was continuously free of disease. His left knee function was virtually normal, with no extension lag and knee flexion of 130° (Fig. 2; Additional file 1: Movie S1). Quadriceps manual muscle test was 5/5. Musculoskeletal Tumor Society (MSTS) score was 30/30 (Enneking et al. 1993).

### Patient 2

A 56-year-old man was referred after unplanned excision of an infrapatellar 35-mm nodule. The pathological diagnosis was clear cell sarcoma. After pre-operative radiotherapy, the same area as in patient 1 was removed because of similar tumor location, BPTB allograft was fashioned to fit the defect, and the allograft was covered by the free contralateral ALT flap. Post-operative course was uneventful. Post-operative management was similar to Patient 1, with the reduced period of the knee brace maintained in straight of 8 weeks, reflecting on the slightly limited ROM in the previous case. Bone union was completed 1 year after surgery. At 48 months post-operatively, the patient was continuously free of disease and his knee function was satisfactory, with minor knee extension lag (<5°), knee flexion of 145°, full muscle strength and MSTS score of 30/30.

### Patient 3

A 28-year-old man was referred after unplanned excision of an infrapatellar 4-cm nodule. The pathologic diagnosis was extraskeletal Ewing’s sarcoma. After neo-adjuvant chemotherapy and radiotherapy, re-excision and reconstruction with BPTB allograft and ALT flap were performed, similar to patients 1 and 2. Post-operative management was similar to the previous 2 cases, with the reduced period of the knee brace maintained in straight of 6 weeks. Post-operative course was uneventful. Bone union was completed 1 year after surgery. At 30 months after surgery, the patient was continuously free of disease. The bone–bone junctions were united, and his knee function was satisfactory, with minor knee extension lag (<5°), knee flexion of 135°, full muscle strength and MSTS score of 30/30.

### Patient 4

**Fig. 3:**
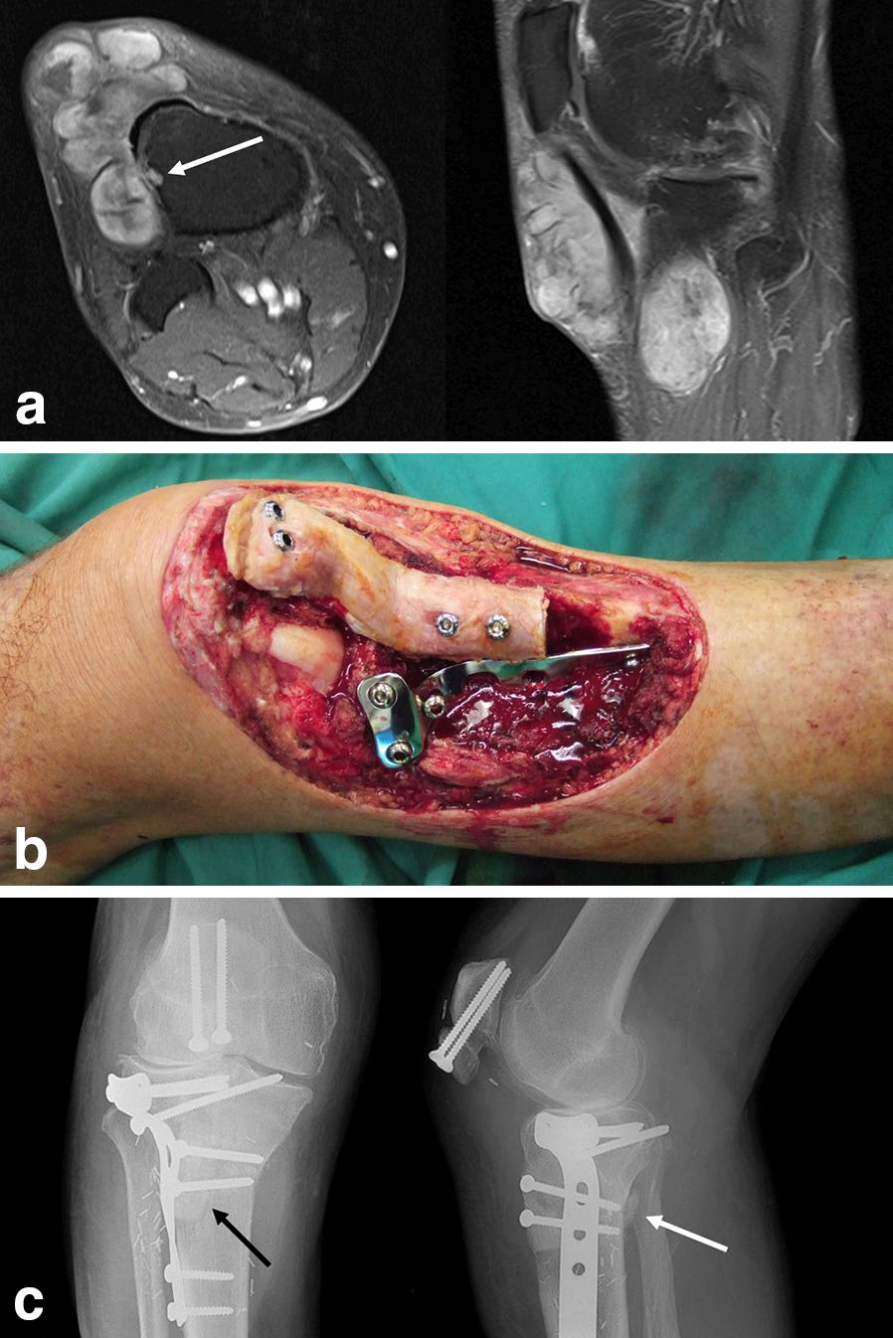
Radiologic studies and photographs of patient 4. **a** T1 gadolinium-enhanced magnetic resonance images of at the first presentation demonstrate partial tumor invasion into the lateral cortex of the proximal tibia (*arrow*). **b** After allograft reconstruction, the proximal tibia was reinforced with a lateral proximal tibial plate and screws. **c** Radiographs show a stress fracture in the proximal tibia 5 months post-operatively. A *black arrow* and *white arrow* point a thin oblique fracture line and posterior fracture-associated callus formation, respectively

A 72-year-old man presented with a 9-cm painless mass in the right knee just lateral to the distal end of patellar tendon. The tumor was located subcutaneously and intra-muscularly but partially invaded into the lateral cortex of the proximal tibia (Fig. 3a). Core needle biopsy confirmed the diagnosis of synovial cell sarcoma. After pre-operative radiotherapy, wide resection included the lower one-third of the patella, the whole patellar tendon, infrapatellar fat pad, anterior lateral knee joint capsule, and one anterior-lateral third in axial section of the proximal tibia extending more distal than the previous cases. After resection, BPTB allograft was fixed to the residual patella and proximal tibia with screws. A lateral proximal tibial plate and screws were added but no cable wire was added because the suitable hole site for cable wiring, just distal to the tibial tuberosity, was resected (Fig. 3b). Post-operatively, we managed this patient similar to the previous patients, maintaining the operated knee straight in splint for the first 8 weeks. Five months post-operatively, an increasingly painful stress fracture of the proximal tibia was diagnosed (Fig. 3c). The fracture was fixed with another plate. Bone union between the implanted allograft and host bones was completed 1 year after surgery. At 18 months after the initial surgery, the fracture site was united, FWB was tolerated, but the knee ROM was restricted with −10° of extension and 115° of flexion. MSTS score was 28/30 (93 %).

## Discussion and evaluation

With advances in surgical techniques, imaging technology, and adjuvant chemotherapy and radiotherapy, approximately 90 % of extremity sarcomas can be treated with limb-salvage surgeries, without sacrificing oncological results (Henshaw and Malawer 2001). However, reconstruction after wide resection for sarcomas involving the patellar tendon can be challenging, with difficulty in obtaining satisfactory function even if the tumor is small. Because of its rarity, only a limited number of single cases using autologous or recycled graft for reconstruction have been published (Table 3) (Muramatsu et al. 2010; Peyser and Makley 1996; Osanai et al. 2008; Nakashima et al. 2012; Machens et al. 2005; Fukui et al. 1999), but to our knowledge the use of BPTB allograft for sarcoma cases has not been reported.

**Table 3:** Previous case reports on patellar tendon reconstruction after sarcoma resections

Author (year)	Resected tissue	Reconstruction	FU	Functional results complication(s)
Muramatsu et al. (2010)	Q, P, PT	Recycling (liquid nitrogen)	18 M	ROM: 0°–10°–110° MMT (Q): 4 +/5
Nakashima et al. (2012)	PT	Fascia lata with iliac bone	3 Y	ROM: 0°–110°
Osanai et al. (2008)	P, PT	Gastrocnemius muscle flap, Achilles tendon	1 Y	Extension lag 5° 85° flexion MSTS score: 90 %
Peyser and Makley (1996)	PT	Biceps tendon-osseous graft, ST tendon, wiring	4 Y	Full function
Machens et al. (2005)	P, PT	Latismus dorsi flap only	3 Y	Extension lag (+) Local recurrence
Fukui et al. (1999)	PT	Hamstring tendons	20 M	No extension lag Full ROM

*Q* quadriceps, *P* patella, *PT* patellar tendon, *ST* semitendinosus, *FU* follow-up, *Y* years, *M* months, *ROM* range of motion, *MMT* manual muscle test, *MSTS* musculoskeletal tumor society

Patellar tendon ruptures associated with TKR, a similar clinical situation requiring re-establishment of knee extension mechanism, are more common and have been well studied. Common reconstruction options include direct suture of tendon tear for acute cases, autologous tendon graft using the hamstrings or gracilis tendon(s), Achilles tendon allograft with calcaneus bone block, and BPTB allograft for re-rupture or chronic cases (Cadambi and Engh 1992; Crossett et al. 2002; Malhotra et al. 2008; Brooks 2009). In the case series of BPTB allograft reconstruction by Malhotra R et al. (Malhotra et al. 2008), both junctions of allograft and host tissue were bone–bone instead of soft tissue-soft tissue, similar to our method, and their results were satisfactory at 14–30 months after patellar tendon reconstruction.

The four cases included in this case series had virtually full knee function without major complications, except for one fracture. The key points of our method include (1) bone–bone junctions between allograft and host tissue, (2) (if possible) reinforcement with a circlage cable wire through the proximal tibia intra-osseously and the quadriceps just above the superior patellar pole, (3) free flap coverage, (4) oblique osteotomy of the inferior patella, and (5) case-by-case post-operative management.

**Fig. 4:**
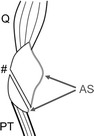
Scheme of patellar osteotomy in this case series. *Q* quadriceps, *PT* patellar tendon, *AS* articular surface, # osteotomy line

The rationale for our approach is as follows. Firstly, bone–bone junction appears superior to soft tissue-soft tissue junction, especially for allograft reconstruction. Indeed, poor long-term functions of quadriceps-patella-patellar tendon-bone allograft were reported (Emerson et al. 1994; Nazarian and Booth 1999). This late failure was thought due to loosening or healing process problems in the proximal quadriceps–quadriceps junction. Secondly, the lack of vascularity of allograft tissue and hypovascularity of irradiated tissue are major concerns for our method, and the durability of BPTB in the setting of reconstruction after radiation is uncertain. In our case series, allograft was covered with a non-irradiated vascularized flap. We speculate that the use of free flap could improve the re-vascularization and internal repair of allograft, although there may still remain a consistent risk of failure. Further follow-up and attention is necessary for possible patellar tendon rupture on a long-term bases. Thirdly, for the first 1–2 years before incorporation and healing of host allograft tissue (Roberts et al. 1991; Muramatsu et al. 2008), circlage cable wire support is thought to be useful to protect the integrity of patellar tendon allograft. A similar use of cable loop for repair protection was reported by Brooks (Brooks 2009). To minimize functional loss and avoid bulky appearance, we chose antero-lateral thigh flap. Fourthly, the degenerative change caused by allograft replacement is predicted to be minimal because the oblique osteotomy of the inferior patella in our method can preserve nearly the whole original cartilage surface of the patella (Fig. 4). Lastly, bone deficit can vary depending on tumor location and may need longer weight-bearing restriction to avoid post-operative fracture as in one patient in our case series. Rosenberg reported 3 cases (6 %) of post-operative fracture at the site of the tibial bone block after allograft reconstruction for TKR-related patellar tendon ruptures (Rosenberg 2012). The risk of post-operative tibial fracture cannot be ignored especially where preoperative radiotherapy is also used which is known to weaken bone.

## Conclusion

In conclusion, we reported a new reconstruction option for patellar tendon deficit after sarcoma resection using BPTB allograft and free flap reconstruction. This novel method can be a viable reconstruction option promising satisfactory clinical outcomes for peri-patellar tendon sarcomas. Long-term restriction of activity including weight-bearing should be considered to prevent post-operative fracture if a larger proportion of proximal tibia is involved in wide resection.

## Consent

Informed consent for publication of individual patient data, including photographs, images and video, was obtained from the patients.

## Additional file

10.1186/s40064-015-1510-9 The movie of patient 1 at 4 years after surgery.

## Abbreviations

BPTB: bone-patellar tendon-bone
TKR: total knee replacement
ROM: range of motion
FWB: full weight bearing
MSTS: musculoskeletal tumor society
ALT: antero-lateral thigh
